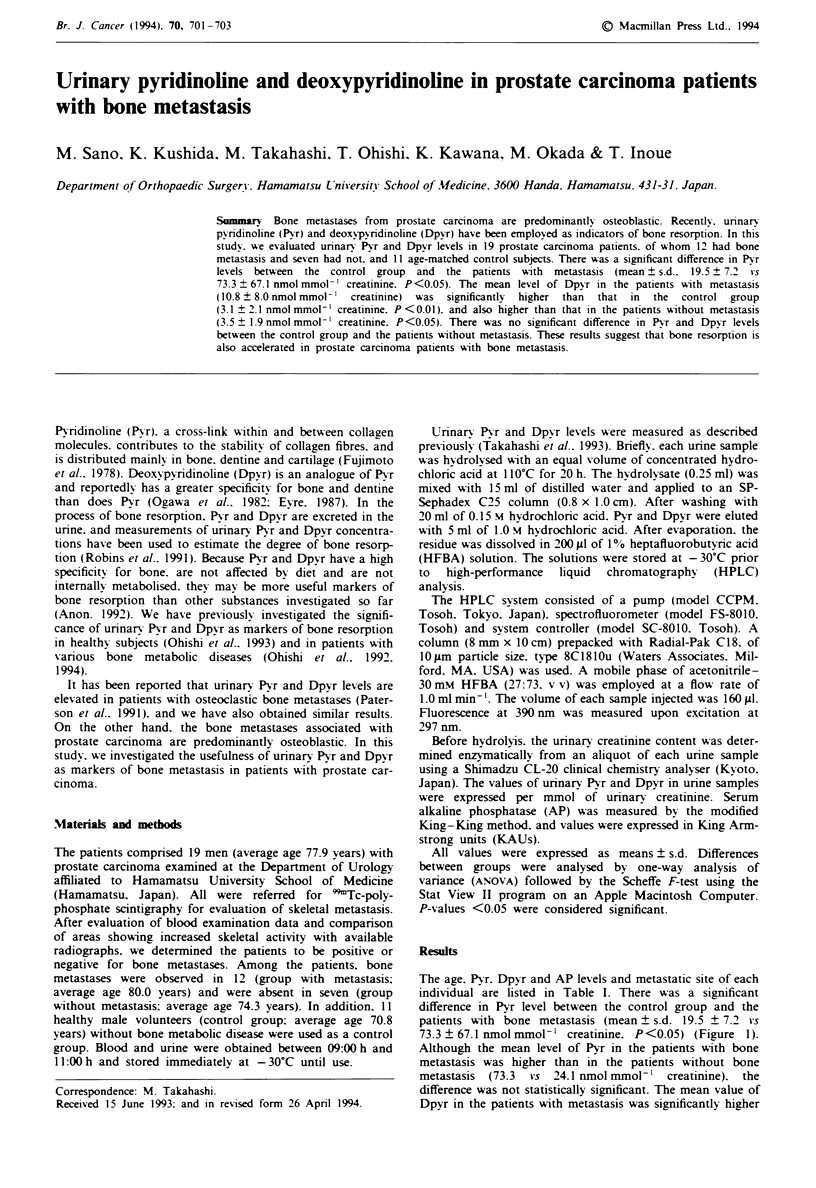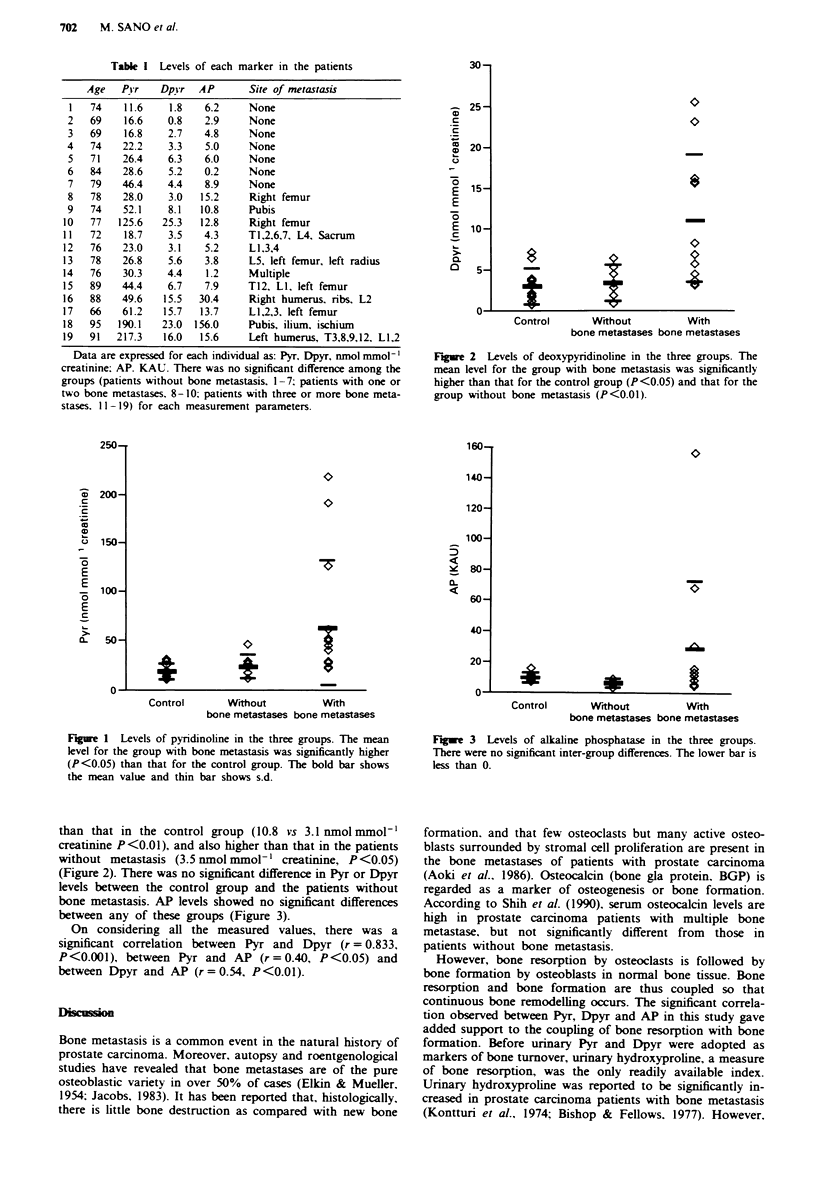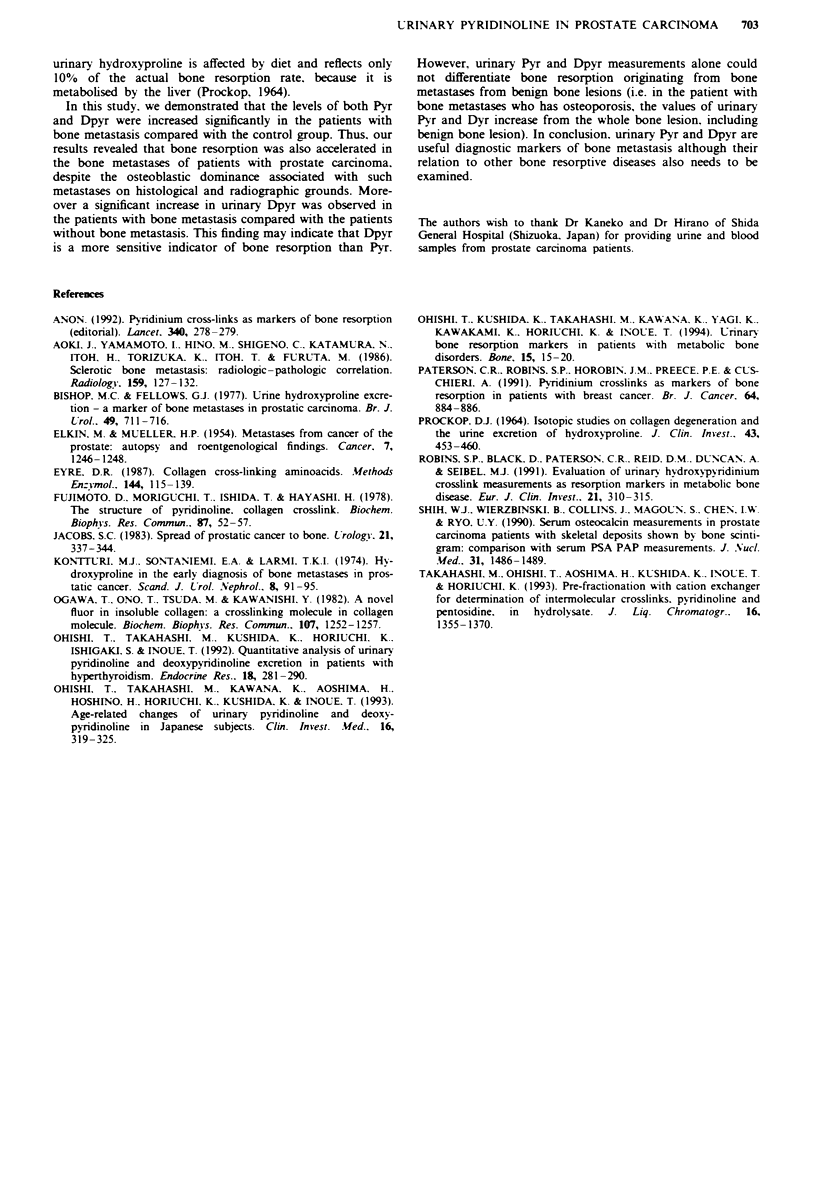# Urinary pyridinoline and deoxypyridinoline in prostate carcinoma patients with bone metastasis.

**DOI:** 10.1038/bjc.1994.377

**Published:** 1994-10

**Authors:** M. Sano, K. Kushida, M. Takahashi, T. Ohishi, K. Kawana, M. Okada, T. Inoue

**Affiliations:** Department of Orthopaedic Surgery, Hamamatsu University School of Medicine, Japan.

## Abstract

Bone metastases from prostate carcinoma are predominantly osteoblastic. Recently, urinary pyridinoline (Pyr) and deoxypyridinoline (Dpyr) have been employed as indicators of bone resorption. In this study, we evaluated urinary Pyr and Dpyr levels in 19 prostate carcinoma patients, of whom 12 had bone metastasis and seven had not, and 11 age-matched control subjects. There was a significant difference in Pyr levels between the control group and the patients with metastasis (mean +/- s.d., 19.5 +/- 7.2 vs 73.3 +/- 67.1 nmol mmol-1 creatinine, P < 0.05). The mean level of Dpyr in the patients with metastasis (10.8 +/- 8.0 nmol mmol-1 creatinine) was significantly higher than that in the control group (3.1 +/- 2.1 nmol mmol-1 creatinine, P < 0.01), and also higher than that in the patients without metastasis (3.5 +/- 1.9 nmol mmol-1 creatinine, P < 0.05). There was no significant difference in Pyr and Dpyr levels between the control group and the patients without metastasis. These results suggest that bone resorption is also accelerated in prostate carcinoma patients with bone metastasis.


Br. J. Cancer (1994), 70, 701-703 © Macmillan Press Ltd., 1994

### Urinary pyridinoline and deoxypyridinoline in prostate carcinoma patients with bone metastasis

M. Sano, K. Kushida, M. Takahashi, T. Ohishi, K. Kawana, M. Okada & T. Inoue

*Department of Orthopaedic Surgery, Hamamatsu University School of Medicine, 3600 Handa, Hamamatsu, 431-31, Japan.*

**Summary** Bone metastases from prostate carcinoma are predominantly osteoblastic. Recently, urinary pyridinoline (Pyr) and deoxypyridinoline (Dpyr) have been employed as indicators of bone resorption. In this study, we evaluated urinary Pyr and Dpyr levels in 19 prostate carcinoma patients, of whom 12 had bone metastasis and seven had not, and 11 age-matched control subjects. There was a significant difference in Pyr levels between the control group and the patients with metastasis (mean ± s.d., 19.5 ± 7.2 *vs* 73.3 ± 67.1 nmol mmol$^{-1}$ creatinine, $P<0.05$). The mean level of Dpyr in the patients with metastasis (10.8 ± 8.0 nmol mmol$^{-1}$ creatinine) was significantly higher than that in the control group (3.1 ± 2.1 nmol mmol$^{-1}$ creatinine, $P<0.01$), and also higher than that in the patients without metastasis (3.5 ± 1.9 nmol mmol$^{-1}$ creatinine, $P<0.05$). There was no significant difference in Pyr and Dpyr levels between the control group and the patients without metastasis. These results suggest that bone resorption is also accelerated in prostate carcinoma patients with bone metastasis.

Pyridinoline (Pyr), a cross-link within and between collagen molecules, contributes to the stability of collagen fibres, and is distributed mainly in bone, dentine and cartilage (Fujimoto *et al.*, 1978). Deoxypyridinoline (Dpyr) is an analogue of Pyr and reportedly has a greater specificity for bone and dentine than does Pyr (Ogawa *et al.*, 1982; Eyre, 1987). In the process of bone resorption, Pyr and Dpyr are excreted in the urine, and measurements of urinary Pyr and Dpyr concentrations have been used to estimate the degree of bone resorption (Robins *et al.*, 1991). Because Pyr and Dpyr have a high specificity for bone, are not affected by diet and are not internally metabolised, they may be more useful markers of bone resorption than other substances investigated so far (Anon. 1992). We have previously investigated the significance of urinary Pyr and Dpyr as markers of bone resorption in healthy subjects (Ohishi *et al.*, 1993) and in patients with various bone metabolic diseases (Ohishi *et al.*, 1992, 1994).

It has been reported that urinary Pyr and Dpyr levels are elevated in patients with osteoclastic bone metastases (Paterson *et al.*, 1991), and we have also obtained similar results. On the other hand, the bone metastases associated with prostate carcinoma are predominantly osteoblastic. In this study, we investigated the usefulness of urinary Pyr and Dpyr as markers of bone metastasis in patients with prostate carcinoma.

### Materials and methods

The patients comprised 19 men (average age 77.9 years) with prostate carcinoma examined at the Department of Urology affiliated to Hamamatsu University School of Medicine (Hamamatsu, Japan). All were referred for $^{99m}$Tc-polyphosphate scintigraphy for evaluation of skeletal metastasis. After evaluation of blood examination data and comparison of areas showing increased skeletal activity with available radiographs, we determined the patients to be positive or negative for bone metastases. Among the patients, bone metastases were observed in 12 (group with metastasis; average age 80.0 years) and were absent in seven (group without metastasis; average age 74.3 years). In addition, 11 healthy male volunteers (control group; average age 70.8 years) without bone metabolic disease were used as a control group. Blood and urine were obtained between 09:00 h and 11:00 h and stored immediately at −30°C until use.

Correspondence: M. Takahashi.
Received 15 June 1993; and in revised form 26 April 1994.

Urinary Pyr and Dpyr levels were measured as described previously (Takahashi *et al.*, 1993). Briefly, each urine sample was hydrolysed with an equal volume of concentrated hydrochloric acid at 110°C for 20 h. The hydrolysate (0.25 ml) was mixed with 15 ml of distilled water and applied to an SP-Sephadex C25 column (0.8 × 1.0 cm). After washing with 20 ml of 0.15 M hydrochloric acid, Pyr and Dpyr were eluted with 5 ml of 1.0 M hydrochloric acid. After evaporation, the residue was dissolved in 200 µl of 1% heptafluorobutyric acid (HFBA) solution. The solutions were stored at −30°C prior to high-performance liquid chromatography (HPLC) analysis.

The HPLC system consisted of a pump (model CCPM, Tosoh, Tokyo, Japan), spectrofluorometer (model FS-8010, Tosoh) and system controller (model SC-8010, Tosoh). A column (8 mm × 10 cm) prepacked with Radial-Pak C18, of 10 µm particle size, type 8C1810u (Waters Associates, Milford, MA, USA) was used. A mobile phase of acetonitrile–30 mM HFBA (27:73, v v) was employed at a flow rate of 1.0 ml min$^{-1}$. The volume of each sample injected was 160 µl. Fluorescence at 390 nm was measured upon excitation at 297 nm.

Before hydrolyis, the urinary creatinine content was determined enzymatically from an aliquot of each urine sample using a Shimadzu CL-20 clinical chemistry analyser (Kyoto, Japan). The values of urinary Pyr and Dpyr in urine samples were expressed per mmol of urinary creatinine. Serum alkaline phosphatase (AP) was measured by the modified King–King method, and values were expressed in King Armstrong units (KAUs).

All values were expressed as means ± s.d. Differences between groups were analysed by one-way analysis of variance (ANOVA) followed by the Scheffe *F*-test using the Stat View II program on an Apple Macintosh Computer. *P*-values <0.05 were considered significant.

### Results

The age, Pyr, Dpyr and AP levels and metastatic site of each individual are listed in Table I. There was a significant difference in Pyr level between the control group and the patients with bone metastasis (mean ± s.d. 19.5 ± 7.2 *vs* 73.3 ± 67.1 nmol mmol$^{-1}$ creatinine, $P<0.05$) (Figure 1). Although the mean level of Pyr in the patients with bone metastasis was higher than in the patients without bone metastasis (73.3 *vs* 24.1 nmol mmol$^{-1}$ creatinine), the difference was not statistically significant. The mean value of Dpyr in the patients with metastasis was significantly higher

Table I Levels of each marker in the patients

| | Age | Pyr | Dpyr | AP | Site of metastasis |
|---|---|---|---|---|---|
| 1 | 74 | 11.6 | 1.8 | 6.2 | None |
| 2 | 69 | 16.6 | 0.8 | 2.9 | None |
| 3 | 69 | 16.8 | 2.7 | 4.8 | None |
| 4 | 74 | 22.2 | 3.3 | 5.0 | None |
| 5 | 71 | 26.4 | 6.3 | 6.0 | None |
| 6 | 84 | 28.6 | 5.2 | 0.2 | None |
| 7 | 79 | 46.4 | 4.4 | 8.9 | None |
| 8 | 78 | 28.0 | 3.0 | 15.2 | Right femur |
| 9 | 74 | 52.1 | 8.1 | 10.8 | Pubis |
| 10 | 77 | 125.6 | 25.3 | 12.8 | Right femur |
| 11 | 72 | 18.7 | 3.5 | 4.3 | T1,2,6,7, L4, Sacrum |
| 12 | 76 | 23.0 | 3.1 | 5.2 | L1,3,4 |
| 13 | 78 | 26.8 | 5.6 | 3.8 | L5, left femur, left radius |
| 14 | 76 | 30.3 | 4.4 | 1.2 | Multiple |
| 15 | 89 | 44.4 | 6.7 | 7.9 | T12, L1, left femur |
| 16 | 88 | 49.6 | 15.5 | 30.4 | Right humerus, ribs, L2 |
| 17 | 66 | 61.2 | 15.7 | 13.7 | L1,2,3, left femur |
| 18 | 95 | 190.1 | 23.0 | 156.0 | Pubis, ilium, ischium |
| 19 | 91 | 217.3 | 16.0 | 15.6 | Left humerus, T3,8,9,12, L1,2 |

Data are expressed for each individual as: Pyr, Dpyr, nmol mmol$^{-1}$ creatinine; AP, KAU. There was no significant difference among the groups (patients without bone metastasis, 1–7; patients with one or two bone metastases, 8–10; patients with three or more bone metastases, 11–19) for each measurement parameters.

Figure 2 Levels of deoxypyridinoline in the three groups. The mean level for the group with bone metastasis was significantly higher than that for the control group ($P<0.05$) and that for the group without bone metastasis ($P<0.01$).

Figure 1 Levels of pyridinoline in the three groups. The mean level for the group with bone metastasis was significantly higher ($P<0.05$) than that for the control group. The bold bar shows the mean value and thin bar shows s.d.

Figure 3 Levels of alkaline phosphatase in the three groups. There were no significant inter-group differences. The lower bar is less than 0.

than that in the control group (10.8 vs 3.1 nmol mmol$^{-1}$ creatinine $P<0.01$), and also higher than that in the patients without metastasis (3.5 nmol mmol$^{-1}$ creatinine, $P<0.05$) (Figure 2). There was no significant difference in Pyr or Dpyr levels between the control group and the patients without bone metastasis. AP levels showed no significant differences between any of these groups (Figure 3).

On considering all the measured values, there was a significant correlation between Pyr and Dpyr ($r = 0.833$, $P<0.001$), between Pyr and AP ($r = 0.40$, $P<0.05$) and between Dpyr and AP ($r = 0.54$, $P<0.01$).

### Discussion

Bone metastasis is a common event in the natural history of prostate carcinoma. Moreover, autopsy and roentgenological studies have revealed that bone metastases are of the pure osteoblastic variety in over 50% of cases (Elkin & Mueller, 1954; Jacobs, 1983). It has been reported that, histologically, there is little bone destruction as compared with new bone formation, and that few osteoclasts but many active osteoblasts surrounded by stromal cell proliferation are present in the bone metastases of patients with prostate carcinoma (Aoki et al., 1986). Osteocalcin (bone gla protein, BGP) is regarded as a marker of osteogenesis or bone formation. According to Shih et al. (1990), serum osteocalcin levels are high in prostate carcinoma patients with multiple bone metastase, but not significantly different from those in patients without bone metastasis.

However, bone resorption by osteoclasts is followed by bone formation by osteoblasts in normal bone tissue. Bone resorption and bone formation are thus coupled so that continuous bone remodelling occurs. The significant correlation observed between Pyr, Dpyr and AP in this study gave added support to the coupling of bone resorption with bone formation. Before urinary Pyr and Dpyr were adopted as markers of bone turnover, urinary hydroxyproline, a measure of bone resorption, was the only readily available index. Urinary hydroxyproline was reported to be significantly increased in prostate carcinoma patients with bone metastasis (Kontturi et al., 1974; Bishop & Fellows, 1977). However, urinary hydroxyproline is affected by diet and reflects only 10% of the actual bone resorption rate, because it is metabolised by the liver (Prockop, 1964).

In this study, we demonstrated that the levels of both Pyr and Dpyr were increased significantly in the patients with bone metastasis compared with the control group. Thus, our results revealed that bone resorption was also accelerated in the bone metastases of patients with prostate carcinoma, despite the osteoblastic dominance associated with such metastases on histological and radiographic grounds. Moreover a significant increase in urinary Dpyr was observed in the patients with bone metastasis compared with the patients without bone metastasis. This finding may indicate that Dpyr is a more sensitive indicator of bone resorption than Pyr. However, urinary Pyr and Dpyr measurements alone could not differentiate bone resorption originating from bone metastases from benign bone lesions (i.e. in the patient with bone metastases who has osteoporosis, the values of urinary Pyr and Dyr increase from the whole bone lesion, including benign bone lesion). In conclusion, urinary Pyr and Dpyr are useful diagnostic markers of bone metastasis although their relation to other bone resorptive diseases also needs to be examined.

The authors wish to thank Dr Kaneko and Dr Hirano of Shida General Hospital (Shizuoka, Japan) for providing urine and blood samples from prostate carcinoma patients.

### References

ANON. (1992). Pyridinium cross-links as markers of bone resorption (editorial). *Lancet*, **340**, 278-279.

AOKI, J., YAMAMOTO, I., HINO, M., SHIGENO, C., KATAMURA, N., ITOH, H., TORIZUKA, K., ITOH, T. & FURUTA, M. (1986). Sclerotic bone metastasis: radiologic-pathologic correlation. *Radiology*, **159**, 127-132.

BISHOP, M.C. & FELLOWS, G.J. (1977). Urine hydroxyproline excretion - a marker of bone metastases in prostatic carcinoma. *Br. J. Urol.*, **49**, 711-716.

ELKIN, M. & MUELLER, H.P. (1954). Metastases from cancer of the prostate: autopsy and roentgenological findings. *Cancer*, **7**, 1246-1248.

EYRE, D.R. (1987). Collagen cross-linking aminoacids. *Methods Enzymol.*, **144**, 115-139.

FUJIMOTO, D., MORIGUCHI, T., ISHIDA, T. & HAYASHI, H. (1978). The structure of pyridinoline, collagen crosslink. *Biochem. Biophys. Res. Commun.*, **87**, 52-57.

JACOBS, S.C. (1983). Spread of prostatic cancer to bone. *Urology*, **21**, 337-344.

KONTTURI, M.J., SONTANIEMI, E.A. & LARMI, T.K.I. (1974). Hydroxyproline in the early diagnosis of bone metastases in prostatic cancer. *Scand. J. Urol. Nephrol.*, **8**, 91-95.

OGAWA, T., ONO, T., TSUDA, M. & KAWANISHI, Y. (1982). A novel fluor in insoluble collagen: a crosslinking molecule in collagen molecule. *Biochem. Biophys. Res. Commun.*, **107**, 1252-1257.

OHISHI, T., TAKAHASHI, M., KUSHIDA, K., HORIUCHI, K., ISHIGAKI, S. & INOUE, T. (1992). Quantitative analysis of urinary pyridinoline and deoxypyridinoline excretion in patients with hyperthyroidism. *Endocrine Res.*, **18**, 281-290.

OHISHI, T., TAKAHASHI, M., KAWANA, K., AOSHIMA, H., HOSHINO, H., HORIUCHI, K., KUSHIDA, K. & INOUE, T. (1993). Age-related changes of urinary pyridinoline and deoxypyridinoline in Japanese subjects. *Clin. Invest. Med.*, **16**, 319-325.

OHISHI, T., KUSHIDA, K., TAKAHASHI, M., KAWANA, K., YAGI, K., KAWAKAMI, K., HORIUCHI, K. & INOUE, T. (1994). Urinary bone resorption markers in patients with metabolic bone disorders. *Bone*, **15**, 15-20.

PATERSON, C.R., ROBINS, S.P., HOROBIN, J.M., PREECE, P.E. & CUSCHIERI, A. (1991). Pyridinium crosslinks as markers of bone resorption in patients with breast cancer. *Br. J. Cancer*, **64**, 884-886.

PROCKOP, D.J. (1964). Isotopic studies on collagen degeneration and the urine excretion of hydroxyproline. *J. Clin. Invest.*, **43**, 453-460.

ROBINS, S.P., BLACK, D., PATERSON, C.R., REID, D.M., DUNCAN, A. & SEIBEL, M.J. (1991). Evaluation of urinary hydroxypyridinium crosslink measurements as resorption markers in metabolic bone disease. *Eur. J. Clin. Invest.*, **21**, 310-315.

SHIH, W.J., WIERZBINSKI, B., COLLINS, J., MAGOUN, S., CHEN, I.W. & RYO, U.Y. (1990). Serum osteocalcin measurements in prostate carcinoma patients with skeletal deposits shown by bone scintigram: comparison with serum PSA PAP measurements. *J. Nucl. Med.*, **31**, 1486-1489.

TAKAHASHI, M., OHISHI, T., AOSHIMA, H., KUSHIDA, K., INOUE, T. & HORIUCHI, K. (1993). Pre-fractionation with cation exchanger for determination of intermolecular crosslinks, pyridinoline and pentosidine, in hydrolysate. *J. Liq. Chromatogr.*, **16**, 1355-1370.